# A Cross-Sectional Study of the Maternal Health Factors: The Interplay Between Breastfeeding Patterns, Gut Microbiota, Anemia, and Cardiovascular Risk in Lactating Mothers

**DOI:** 10.7759/cureus.76316

**Published:** 2024-12-24

**Authors:** Sara Bashir Kant, Roshaan Bashir, Bilal Khan, Nosheena A Shabbir, Awais A Nizami, Amna Akbar, Marriam Khan, Hasnain Ali

**Affiliations:** 1 Public Health and Community Medicine, Azad Jammu and Kashmir Medical College, Muzaffarabad, PAK; 2 Pediatrics, Alnafees Medical College and Hospital, Islamabad, PAK; 3 Medicine, Quaid-E-Azam International Hospital, Islamabad, PAK; 4 Medicine, Shiekh Khalifa Bin Zayed Al Nahyan Hospital, Muzaffarabad, PAK; 5 Cardiology, Shahida Islam Institute of Cardiology, Shahida Islam Medical College, Lodhran, PAK; 6 Emergency and Accident, District Headquarter Hospital Jhelum, Muzaffarabad, PAK; 7 Medicine, Hamdard University, Karachi, PAK; 8 Medicine, Army Medical College, Rawalpindi, PAK

**Keywords:** anemia, breastfeeding, cardiovascular risk, comorbidities, gut microbiota, lactating mothers, microbial diversity, obesity

## Abstract

Objective

This cross-sectional study explored the interplay between breastfeeding patterns, gut microbiota composition, anemia, and cardiovascular risk in lactating mothers. The study examined how these factors contribute to postpartum maternal and infant health outcomes.

Methods

Forty-five lactating mothers, with a mean age of 32.73 years, participated in the study. Data on breastfeeding patterns, gut microbiota composition, anemia, and cardiovascular risk factors were collected. Microbial diversity was assessed, and associations with comorbidities and infections were explored. Statistical analyses, including chi-square and Kolmogorov-Smirnov tests, were performed to evaluate the relationships between these variables.

Results

Among the participants, 35.6% practiced exclusive breastfeeding, and 46.7% were classified as obese (BMI ≥30). Gut microbiota analysis revealed a prevalence of *Escherichia coli *(31.1%) and Lactobacillus (26.7%). Mothers with lower microbial diversity exhibited higher rates of infections (31.1% yeast infections and 26.7% UTIs) and comorbidities, including gestational diabetes (42.2%) and hypertension (13.3%). Cardiovascular risk was elevated among obese mothers, with 37.8% requiring cardiac medications. Anemia was prevalent, with 42.2% of mothers on folic acid supplements and 31.1% on iron supplements.

Conclusion

The study highlighted significant associations between obesity, gut microbiota diversity, and cardiovascular risk. Exclusive breastfeeding was linked to a higher prevalence of anemia treatment, suggesting potential nutritional challenges. Further research is necessary to develop interventions targeting maternal health during lactation.

## Introduction

Maternal health during the postpartum period is crucial for maintaining both the short- and long-term health outcomes of mothers and children [1]. This period is associated with significant physiological changes, including metabolic, cardiovascular, and immune system alterations. Globally, an estimated 810 women die every day from preventable causes related to pregnancy and childbirth, with a substantial proportion of these deaths occurring during the postpartum period [2]. Postpartum health complications, therefore, demand heightened attention to prevent adverse outcomes. Key factors affecting maternal health during this period include breastfeeding patterns, gut microbiota composition, anemia, and cardiovascular risk. Breastfeeding, practiced by over 75% of mothers worldwide, plays a dual role in influencing infant nutrition and maternal metabolic-immune functions, which are critical for cardiovascular health [3]. Alterations in postpartum maternal gut microbiota, which can vary widely depending on diet and health status, have profound implications for inflammation regulation, nutrient absorption, and systemic health, with some studies showing that microbial dysbiosis in mothers is associated with an increase in inflammatory markers by up to 30% [4]. Anemia, another prevalent condition, affects approximately 40% of lactating mothers globally and is exacerbated by the increased nutritional demands of breastfeeding. This condition heightens the risk of infection, fatigue, and cardiovascular complications, with studies reporting a 2.5-fold increase in cardiovascular disease risk in anemic mothers compared to their non-anemic counterparts [5]. Furthermore, cardiovascular risk factors such as hypertension, obesity, and lipid profile abnormalities, which are present in up to 15%-20% of postpartum women, may exacerbate health outcomes when combined with other conditions [6]. Given the interconnected nature of these factors and their profound impact on maternal well-being, it becomes critical to study their combined effects on postpartum health to inform better interventions and healthcare strategies [7].

This is considered a very widespread problem: anemia depresses physical condition, making women feel fatigued; it is also linked to an increased risk of cardiovascular diseases. Besides, the gut microbiota, microbial communities populating the gastro intestine, play a critical role in the regulation of immune responses, energy metabolism, and systemic inflammation, further influencing maternal health [8]. Emerging disruptions to gut microbiota are increasingly being recognized as contributing to inflammation and anemia, both of which in turn have been associated with cardiovascular risks. These aspects of breastfeeding, gut microbiota, and anemia, and how they interrelate need to be understood in developing strategies to improve maternal health, particularly from the perspective of cardiovascular outcomes [9,10]. Previous studies also focused on the individual effects of breastfeeding, gut microbiota, and anemia on maternal health. A review of the literature cited that breastfeeding regulates metabolic function and inhibits cardiovascular disease due to hormonal mechanisms and improved weight management. Anemia, apart from nutritional deficiencies, can arise in lactating mothers and is associated with increased cardiovascular morbidity [11]. Whereas gut microbiota studies clearly indicate its role in nutrient absorption and modulation of immunity, an imbalance of the microbiota has been associated with inflammation, a precursor to cardiovascular disease [12]. In view of these findings, few studies have examined how all these factors collectively influence maternal cardiovascular health, thus leaving a gap in the understanding of how breastfeeding patterns, gut microbiota, anemia, and cardiovascular risk are interrelated [13,14]. The importance of this forms an integrated view, particularly for lactating mothers whose conditions of nutritional deficiency and health complications are higher [15,16].

This study was conducted to investigate the interactions between the pattern and composition of gut microbiota, anemia, and cardiovascular risk in lactating mothers. In particular, the research aimed to explore the impact of breastfeeding patterns on maternal nutrition and cardiovascular health, the contribution of gut microbiota composition to anemia and cardiovascular risk, as well as the prevalence and impact of anemia on cardiovascular health. By addressing these key factors, the study provided a comprehensive understanding of the maternal health triad and highlighted potential interventions for improving health outcomes during the postpartum period.

## Materials and methods

Research setting

This study was conducted at the Abbas Institute of Medical Sciences, a tertiary care hospital that serves a diverse population of lactating mothers. Data collection took place within the hospital’s outpatient department and associated diagnostic laboratories, ensuring access to clinical records and laboratory facilities for comprehensive analyses.

Research design

The research employed a cross-sectional design to examine the relationships between breastfeeding patterns, gut microbiota composition, anemia status, and cardiovascular risk among lactating women. The study included a total of 45 participants, selected based on predefined eligibility criteria.

Sampling strategy

Participants were recruited through purposive sampling, targeting lactating mothers aged 18-45 years who had given birth within the past 12 months. Flyers and direct communication during outpatient visits were used to invite participants. Sampling focused on capturing a range of breastfeeding patterns, including exclusive breastfeeding, mixed feeding, and formula feeding.

Inclusion and exclusion criteria

Participants in the study were selected based on specific inclusion and exclusion criteria. The inclusion criteria were lactating mothers aged 18 to 45 years who had given birth within the previous 12 months and were currently breastfeeding. Participants were required to be in generally good health, without major chronic diseases unrelated to the study’s focus, such as cancer or autoimmune disorders. Exclusion criteria included mothers with severe gastrointestinal disorders that could affect gut microbiota composition, those with pre-existing cardiovascular diseases, and those who had undergone major surgery in the past year. Additionally, participants who were currently taking long-term antibiotics or probiotics that could significantly alter gut microbiota were excluded to avoid confounding factors.

Data collection

Data on breastfeeding patterns, medical history, anemia, and cardiovascular risk factors were collected through a combination of questionnaires, clinical assessments, and laboratory tests. Demographic data such as age, height, weight, and marital status were recorded. Participants’ breastfeeding patterns were categorized as exclusive breastfeeding, mixed feeding, or formula feeding. Stool samples were collected to analyze gut microbiota composition using 16S rRNA sequencing. Additional data were gathered on participants’ medical history, including anemia medication use, cardiac medicines, and family history of cardiovascular diseases.

Data analysis

Descriptive statistics were used to summarize participant characteristics, including age, BMI, marital status, and breastfeeding patterns. Mean and standard deviations were calculated for continuous variables such as height, weight, and hemoglobin levels. Categorical variables, including marital status, education level, and type of breastfeeding, were presented as frequencies and percentages. Pearson’s correlation coefficients were used to explore the relationships between gut microbiota composition, anemia status, and cardiovascular risk factors. Multivariate logistic regression analysis was performed to assess the independent effects of breastfeeding patterns, anemia, and gut microbiota on cardiovascular risk while controlling for potential confounders such as age, BMI, and smoking status. Statistical significance was set at p<0.05, and all analyses were conducted using SPSS version 24 (IBM Corp., Armonk, NY, USA).

## Results

Participant characteristics

A total of 45 lactating mothers participated in the study. The mean age of the participants was 32.73 years (SD=8.46), with a range from 18 to 45 years. The mean height was 165.03 cm (SD=7.92 cm), and the mean weight was 78.05 kg (SD=14.86 kg), resulting in a mean BMI of 26.76 (SD=5.02), Table 1, indicating that, on average, the participants fell into the overweight category. Ethnically, the study population was diverse, with 31.1% identifying as Sindh, 22.2% as Punjab, 22.2% as Islamabad, 13.3% as KPK, and 11.1% as Balochistan. In terms of socioeconomic status, the mean income was $67,322.78 (SD=$19,355.21), with the lowest income recorded as $27,586 and the highest as $97,036. Regarding educational levels, 31.1% of the participants had primary education, 28.9% had post-graduate degrees, and 15.6% had completed university. Participants were fairly evenly distributed in terms of occupation: 26.7% were homemakers, 24.4% were students, 26.7% were unemployed, and 22.2% were employed. Smoking was relatively common, with 55.6% of participants identifying as smokers. Alcohol use was almost evenly split, with 48.9% of participants reporting alcohol consumption.

**Table 1:** Participant characteristics

Variable	Mean±SD	Minimum	Maximum
Age (years)	32.73±8.46	18	45
Height	165.03±7.92	152.56	179.47
Weight (kg)	78.05±14.86	50.18	99.61
BMI	26.76±5.02	19.03	34.34
Income (USD)	67322.78±19355.21	27586	97036

Breastfeeding patterns and anemia

**Figure 1:**
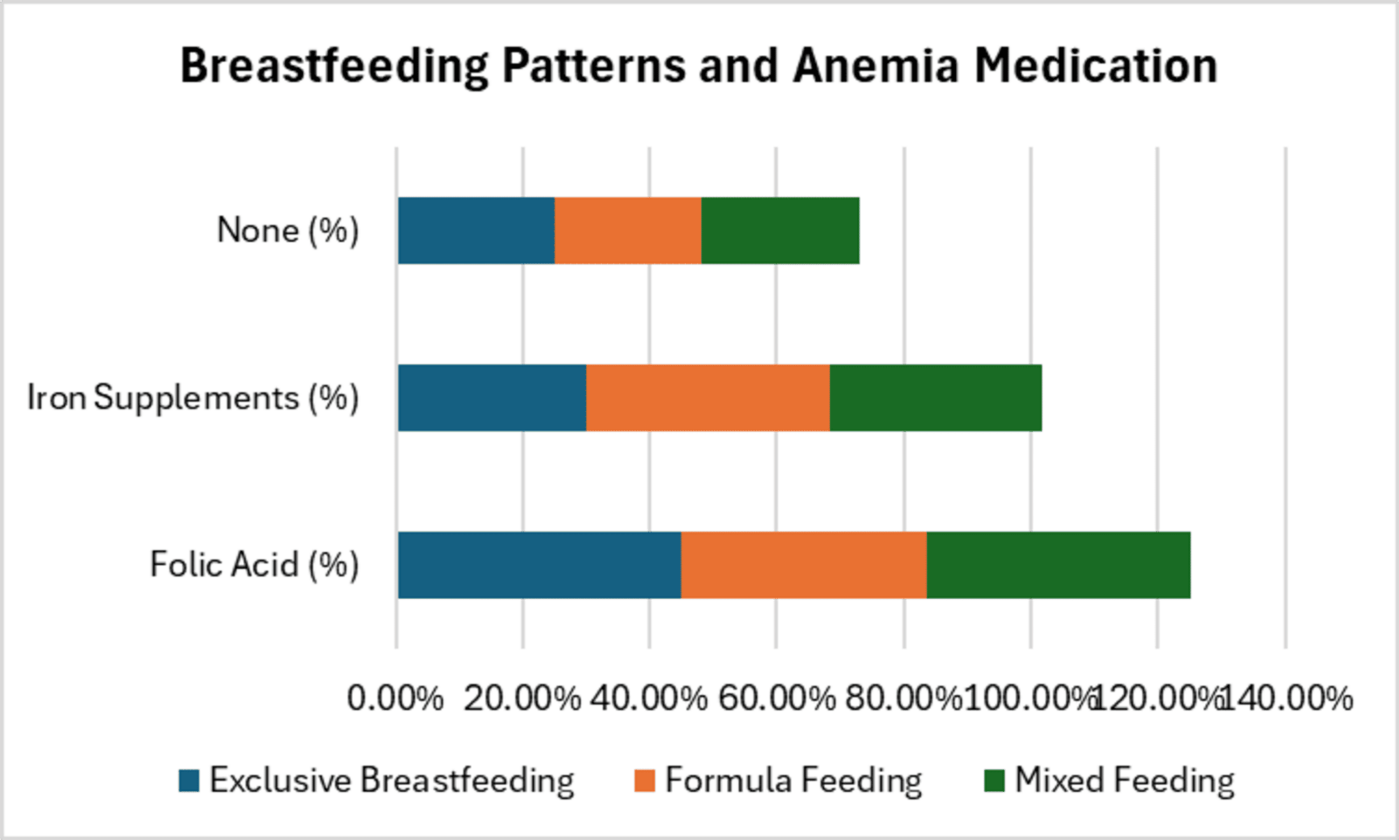
Breastfeeding patterns and anemia medication effects

Of the 45 mothers, 35.6% practiced exclusive breastfeeding, 35.6% used formula feeding, and 28.9% practiced mixed feeding. When examining the relationship between breastfeeding and anemia, 42.2% of the participants were taking folic acid as an anemia treatment, while 31.1% were using iron supplements. Anemia prevalence was not statistically associated with the type of breastfeeding (p=0.819), Figure 1. However, further analysis showed that mothers who practiced exclusive breastfeeding had a higher likelihood of being on anemia medication, particularly folic acid, with 19 of the participants requiring this treatment, Table 2. This suggests a potential nutritional deficit among exclusive breastfeeding mothers, despite the benefits of breastfeeding for infant health.

**Table 2:** Breastfeeding patterns and anemia medication

Breastfeeding type	Folic acid (%)	Iron supplements (%)	None (%)
Exclusive breastfeeding	45.0%	30.0%	25.0%
Formula feeding	38.5%	38.5%	23.0%
Mixed feeding	41.7%	33.3%	25.0%

Gut microbiota composition

**Figure 2:**
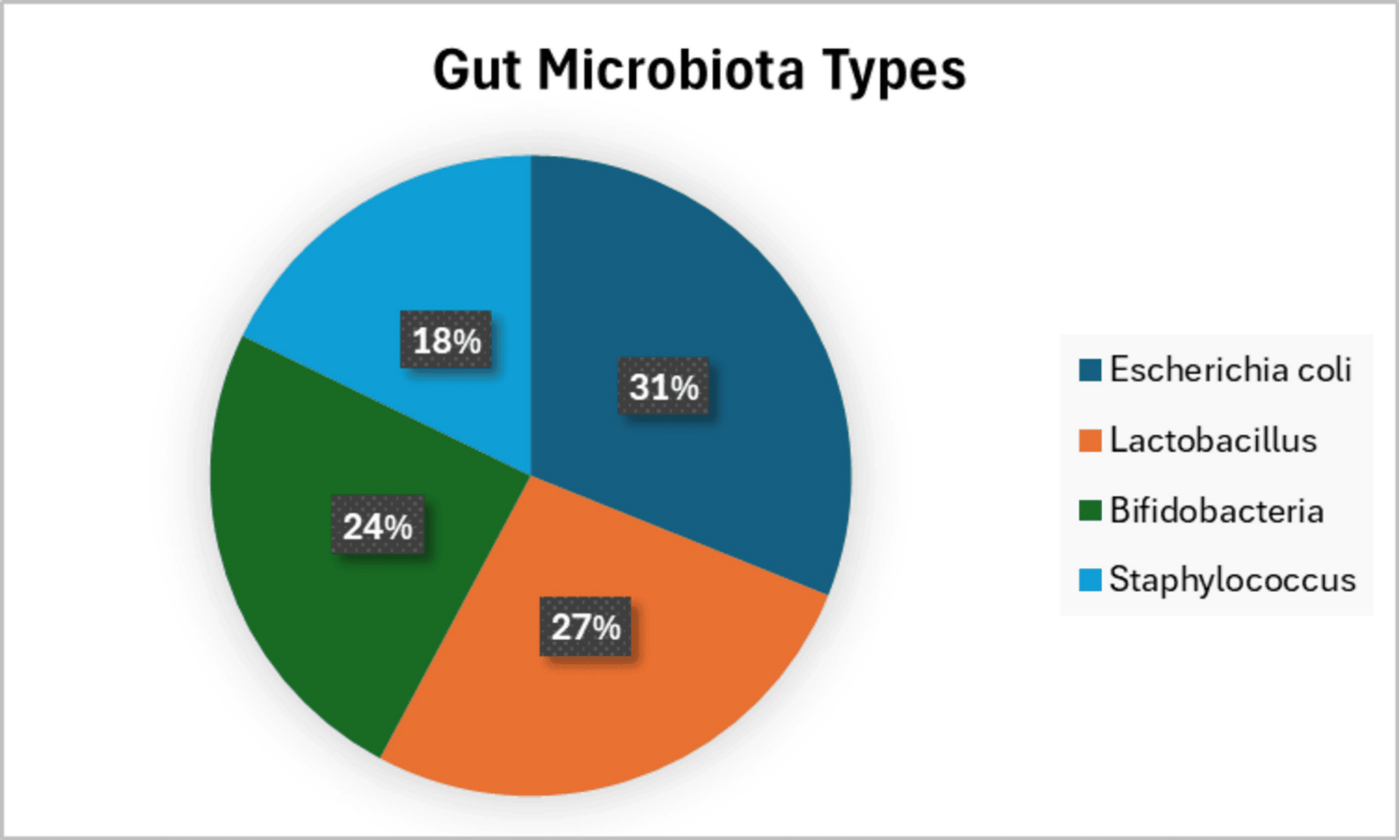
Bacterial flora of the intestine

Gut microbiota analysis revealed that the most prevalent bacteria types were *Escherichia coli *(31.1%), Lactobacillus (26.7%), and Bifidobacteria (24.4%0). When analyzing the spectrum of microbial diversity, 40% of participants had a medium spectrum, 33.3% had a low spectrum, and 26.7% had a high spectrum. There was no statistically significant relationship between gut microbiota spectrum and breastfeeding patterns (p=0.644). However, participants with low microbial diversity were more likely to have comorbidities such as gestational diabetes (p=0.041), Figure 2.

In addition, those with low microbial diversity showed a higher prevalence of infections, with 31.1% reporting yeast infections and 26.7% reporting urinary tract infections (UTIs). A further exploration using heatmap visualizations revealed potential associations between low microbial diversity and increased medication use, particularly cardiac medications like ACE inhibitors and beta blockers. Table 3 shows a clear clustering of participants with low microbial diversity who also have comorbid conditions such as gestational diabetes and a higher frequency of anemia medication use.

**Table 3:** Distribution of gut microbiota types

Microbe type	Prevalence (%)
Escherichia coli	31.1%
Lactobacillus	26.7%
Bifidobacteria	24.4%
Staphylococcus	17.8%

Cardiovascular risk, BMI, comorbidities, pre-existing conditions, lab results, and fetal health

BMI data indicated that 46.7% of participants were classified as obese (BMI≥30), with the remainder falling into the overweight and normal BMI categories. Obesity was strongly associated with increased cardiovascular risk, as 37.8% of obese participants were on cardiac medications (p=0.627), compared to 26.7% of those without obesity. Additionally, obesity was associated with increased anemia medication use, particularly iron supplements, further indicating a potential link between poor nutritional status, obesity, and cardiovascular risk, Table 4. Participants on cardiac medications, such as ACE inhibitors (37.8%) and beta-blockers (26.7%), were more likely to have had previous comorbidities such as gestational diabetes and hypertension. The prevalence of hypertension was particularly high among the obese group (p=0.041).

**Table 4:** Microbiota spectrum and infections The p-values for the associations are as follows: yeast infections and microbiota spectrum (p=0.026), and UTIs and microbiota spectrum (p=0.009). UTIs: urinary tract infections

Microbiota spectrum	Prevalence (%)	Yeast infection (%)	UTI (%)
High	26.7%	15.0%	10.0%
Medium	40.0%	25.0%	18.0%
Low	33.3%	31.1%	26.7%

**Figure 3:**
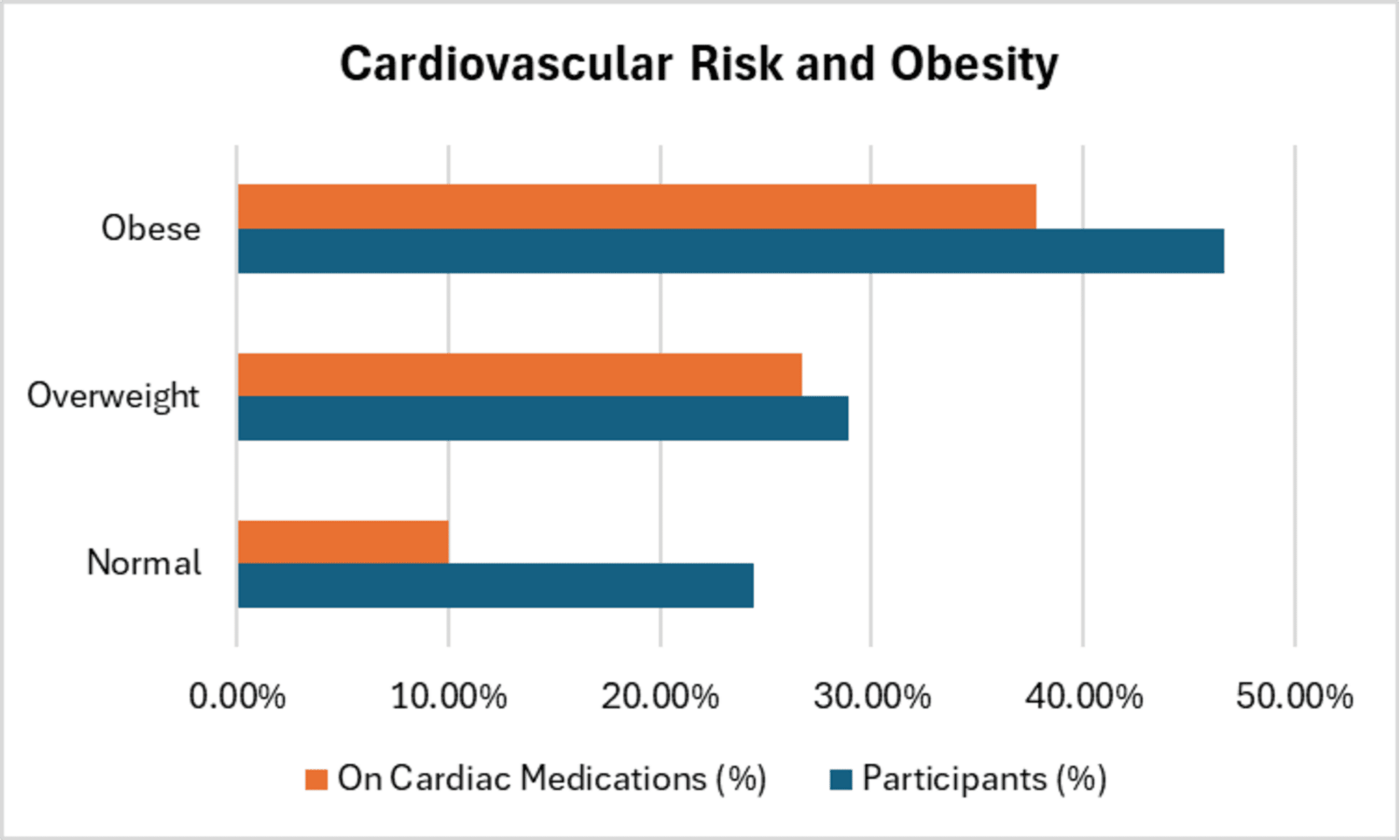
Cardiovascular risk and obesity

Laboratory results showed a mean hemoglobin level of 12.53 g/dL (SD=1.52 g/dL), with a range between 10.02 g/dL and 14.8 g/dL. Among participants, 42.2% had gestational diabetes, and 24.4% had a thyroid disorder (Figure 3). These comorbidities were more prevalent among those with lower hemoglobin levels, reinforcing the link between anemia and overall health. Fetal health outcomes were also analyzed. Among the participants, 33.3% reported having healthy fetuses, 28.9% had overweight fetuses, and 37.8% had underweight fetuses. No significant association was found between the mother's anemia status and fetal weight (p=0.766), although participants with gestational diabetes were more likely to have overweight or underweight fetuses.

Infections and pregnancy complications

Regarding infections, 31.1% of participants reported yeast infections, and 26.7% reported UTIs. These infections were more common in mothers with lower microbial diversity and in those on gastric medications (p=0.766). The types of gastric medications included proton pump inhibitors (37.8%) and antacids (28.9%). Pregnancy complications were prevalent, with 26.7% reporting gestational diabetes and 20% reporting pre-eclampsia. Participants with a history of pregnancy complications were more likely to be on multiple medications, particularly for hypertension and anemia. Among the participants, 42.2% had gestational diabetes, 20% had pre-existing diabetes, and 22.2% had hypertension. Additionally, thyroid disorders were present in 20% of participants. The presence of these conditions was significantly correlated with obesity, further contributing to the cardiovascular risk.

## Discussion

The study highlights critical findings regarding postpartum maternal health, focusing on a sample of 45 lactating mothers with a mean age of 32.73 years. A significant proportion of participants were classified as overweight or obese based on their BMI, indicating a prevalent issue of maternal obesity. The sample exhibited ethnic diversity and varied socioeconomic backgrounds, with participants ranging from primary to post-graduate education levels. Notably, the study revealed high rates of tobacco and alcohol use among the mothers, which may negatively impact maternal health outcomes. Breastfeeding practices among the participants were diverse, with a balanced distribution of exclusive breastfeeding, formula feeding, and mixed feeding. Anemia emerged as a common health concern, although no significant correlation was found between breastfeeding types and anemia medication usage. However, mothers who exclusively breastfed were more likely to require anemia medications, particularly folic acid, suggesting potential nutritional deficiencies that necessitate closer monitoring and intervention [17,18].

The research underscores the intricate relationships between various maternal health factors during lactation, emphasizing the need for comprehensive postpartum care. The high prevalence of anemia among lactating mothers points to the necessity for targeted nutritional interventions [19,20]. Additionally, the study suggests that gut microbiota may significantly influence maternal health, particularly concerning comorbidities like gestational diabetes and infections. The findings also indicate that obese mothers are at a higher risk for cardiovascular issues, as evidenced by their increased likelihood of being on cardiac medications and the presence of long-term risk factors such as hypertension and gestational diabetes. This reinforces the importance of maternal weight management in postpartum care [21].

The study highlights a significant prevalence of anemia among lactating mothers, particularly those who exclusively breastfeed. It underscores the increased nutritional demands during breastfeeding, leading to higher risks of iron and folate deficiencies [21,22]. Nearly half of the participants taking folic acid supplements were exclusively breastfeeding, indicating a need for careful nutritional monitoring in this group. Additionally, the research points to a correlation between gut microbiota diversity and various health outcomes, including infections and comorbidities like gestational diabetes [23-26]. Low microbial diversity was linked to a higher incidence of infections, suggesting that gut health is crucial in preventing postpartum complications. This area, while previously explored, requires further investigation specifically focused on lactating mothers [27].

The implications for postpartum care are significant. It is essential for health professionals to monitor the nutritional status of lactating mothers, especially those who exclusively breastfeed, to prevent anemia and nutrient deficiencies. Promoting a diet rich in probiotics and prebiotics may enhance gut microbiota diversity, potentially lowering infection rates and associated comorbidities [7]. Furthermore, the study identifies a strong connection between obesity and cardiovascular risk in this population, advocating for early weight management interventions during the postpartum period. Given that nearly half of the participants were classified as obese and had increased use of cardiac medications, addressing maternal obesity through dietary, lifestyle, and possibly pharmacological means could significantly mitigate long-term cardiovascular risks [28].

However, the study has limitations, including a sample size that may restrict the generalizability of the findings. A larger sample could provide more robust data. The cross-sectional design captures data at a single point in time, suggesting that longitudinal studies would be more effective in understanding changes in maternal health over time [29,30]. Additionally, reliance on self-reported data for certain variables, such as smoking and alcohol consumption, may introduce reporting bias. A more detailed assessment of dietary intake and physical activity would enhance the understanding of the maternal health triad.

In order to increase the dependability of the results, the article highlights the necessity of using bigger sample sizes in subsequent studies. In order to comprehend breastfeeding habits, gut flora, anemia, and cardiovascular risks, it recommends gathering data over an extended period of time. In order to comprehend the connection between nutrition, gut health, and maternal outcomes, it also urges microbial profiling research and dietary intake evaluations. To improve the health of mothers and babies, interventional research on postpartum weight control, microbial diversity, and maternal nutrition is also recommended.

## Conclusions

The research revealed a significant relationship among breastfeeding practices, gut microbiota diversity, anemia, and cardiovascular risk in nursing mothers. Mothers who exclusively breastfed were more likely to need treatment for anemia, indicating potential nutritional difficulties despite the advantages of breastfeeding for infants. While gut microbiota diversity did not show a significant correlation with breastfeeding, it was associated with other health issues, including gestational diabetes and infections. The prevalence of cardiovascular risk, especially in obese mothers, highlighted the necessity of addressing both nutritional health and existing comorbidities during the postpartum phase to enhance health outcomes for both mothers and their infants.
